# Activated PI3Kδ syndrome in inborn errors of immunity: diagnostic strategies and clinical challenges

**DOI:** 10.3389/fimmu.2025.1735023

**Published:** 2026-01-08

**Authors:** Selcen Bozkurt, Necmiye Ozturk, Melek Yorgun Altunbas, Salim Can, Razin Amirov, Ramin Mahmudov, Burkay Cagan Colak, Esra Karabiber, Manuela Baronio, Vassilios Lougaris, Giulio Tessarin, Sevgi Bilgic-Eltan, Ahmet Ozen, Safa Baris, Elif Karakoc-Aydiner

**Affiliations:** 1 Marmara University, Faculty of Medicine, Department of Pediatrics, Division of Allergy and Immunology, Istanbul Jeffrey Modell Diagnostic and Research Center for Primary Immunodeficiencies, The Isil Berat Barlan Center for Translational Medicine, Immune Deficiency Research and Application Center, European Academy of Allergy and Clinical Immunology Marmara University Hospital Center of Excellence, Istanbul, Türkiye; 2 Marmara University, Faculty of Medicine, Department of Chest Diseases, Division of Adult Allergy and Immunology, Istanbul, Türkiye; 3 Pediatrics Clinic and Institute for Molecular Medicine “A. Nocivelli”, Department of Clinical and Experimental Sciences, University of Brescia, and Azienda Socio-Sanitaria Territoriale (ASST) Spedali Civili di Brescia, Brescia, Italy

**Keywords:** activated phosphoinositide 3-kinase delta syndrome, APDS, diagnostic strategies, inborn errors of immunity, PIK3CD, PIK3R1

## Abstract

**Introduction:**

This study aims to present in a large real-world cohort a diagnostic algorithm developed to facilitate the early recognition of Activated Phosphoinositide 3-Kinase Delta Syndrome (APDS), a rare disease with targeted treatment options, and to provide clinicians with a practical roadmap for navigating diagnostic challenges.

**Methods:**

The study was conducted as a retroactive cross-sectional observational study. We reviewed the medical records of 6,458 pediatric and adult patients who were referred to our clinic between 2018 and 2025. A medical algorithm was generated based on major clinical and laboratory features of APDS. Next-generation sequencing analyses were performed on patients who were appropriate for further evaluation. Variant analysis using in silico predictors and S6 phosphorylation analysis in patients carrying previously undescribed variants were conducted accordingly.

**Results:**

In this cohort of 6,458 patients, the diagnostic algorithm identified 1,138 who met at least one major clinical or laboratory criterion. After excluding 7 with a prior APDS diagnosis and 573 with other inborn errors of immunity, genetic analysis was performed in 20 consenting patients under clinical follow-up (11 [55%] female, 9 [45%] male; median age 15 years; IQR 7.5–24). APDS type 2 was confirmed in 1 patient; five others harbored novel variants of uncertain significance.

**Conclusion:**

Delayed diagnosis and treatment of APDS may result in life-threatening complications and irreversible end-organ damage. Given its heterogeneous, overlapping phenotype, timely referral for genetic testing is essential.

## Introduction

Activated phosphoinositide 3-kinase delta syndrome (APDS) is a rare inborn error of immunity caused by a heterozygous gain-of-function mutation in the catalytic p110δ (*PIK3CD*) subunit or a heterozygous loss-of-function mutation in the regulatory p85α (*PIK3R1*) subunit of the phosphoinositide 3-kinase delta (PI3Kδ) pathway (1–3). It typically presents in early life with recurrent infections, lymphoproliferation, and an increased risk of autoimmune diseases. Over time, serious complications such as bronchiectasis, impaired lung function, chronic diarrhea, and lymphoma may develop (1, 2, 4, 5).

The p110δ and p85α subunits of PI3Kδ play a critical role in the PI3K/Akt signaling pathway, regulating lymphocyte growth and differentiation (1, 2, 4, 6). Dysregulation of this pathway can lead to impaired function of T and B cells. Patients may present with various clinical phenotypes, including agammaglobulinemia, Hyper IgM syndrome, common variable immunodeficiency, and combined immunodeficiency (1, 2, 4, 7). Immunological profiling frequently reveals decreased CD4^+^ T lymphocytes, increased CD8^+^ T lymphocytes, reduced naïve B cells, and class-switch recombination defects (1, 2, 4–7). To demonstrate increased activation of the PI3K-AKT-mTOR signaling pathway in B and T lymphocytes, phosphorylation levels of S6 and AKT proteins can be analyzed. However, standardization of these assays remains challenging, and their use in routine clinical practice is limited. Therefore, a definitive diagnosis requires genetic analysis (2, 5, 8–11).

Followed by the confirmed diagnosis of APDS, antibacterial and antiviral prophylaxis along with immunoglobulin replacement therapy (IgRT) are used to control infections, while sirolimus can be administered to manage lymphoproliferation (2, 4, 8). Since these are not curative or targeted therapies and therefore do not correct the underlying pathogenesis of the disease, curative or targeted therapeutic approaches such as hematopoietic stem cell transplantation or leniolisib should be weighted for APDS patients (2, 8, 12). In recent years, advancements in targeted therapies with leniolisib, a kinase inhibitor that blocks the overactive PI3Kδ protein, have shown potential to improve the quality of life of APDS patients with better safety profiles and fewer adverse effects (2, 13, 14).

The rarity of APDS, combined with its heterogeneous clinical presentation and limited clinician awareness, makes early diagnosis challenging, often leading to delays (1, 8). Given the benefits of targeted therapies and their increasing availability, early diagnosis and timely initiation of treatment are critical to preventing complications and subsequent end-organ damage or death (15). Nevertheless, studies on comprehensive diagnostic approaches capable of distinguishing APDS from other primary antibody deficiencies and immune dysregulatory conditions, particularly those applicable to large and heterogeneous patient populations, remain limited. To address this gap, our study presents a comprehensive diagnostic algorithm applied to a large cohort evaluated for inborn errors of immunity. Our aim is to provide both a practical clinical tool and to share our experiences regarding real-world diagnostic challenges.

## Materials and methods

**Figure 1:**
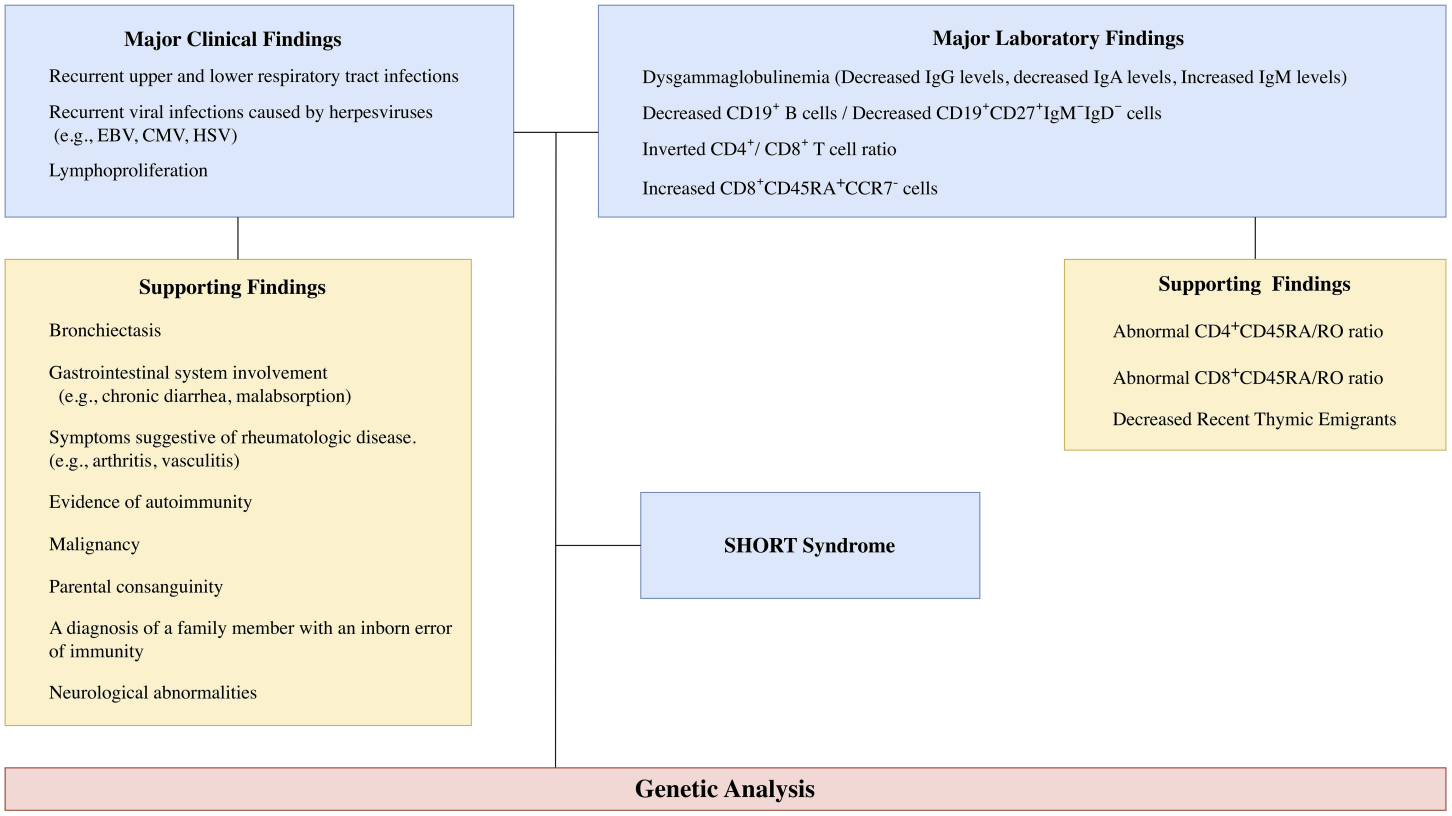
Diagnostic algorithm for Activated PI3Kδ Syndrome (APDS). The prominent clinical and immunologic features of APDS are depicted schematically. Patients presenting with at least one major clinical and laboratory finding were included in APDS screening. immunoglobulin (Ig).

The study was conducted with a retroactive, cross-sectional, observational design. A total of 6,458 pediatric and adult patients who were referred to our clinic between 2018 and 2025 and evaluated for primary antibody deficiencies and immune dysregulation were included in the study. All participants were assessed according to the diagnostic features of APDS, encompassing clinical and laboratory findings (2, 4, 8, 11). Patients with a prior diagnosis of APDS who were under follow-up, and those given alternative diagnoses according to the European Society for Immunodeficiencies (ESID) criteria or the Middle East and North Africa Diagnosis and Management (MENA) Guidelines, were excluded (16, 17). Clinical manifestations observed in at least 50% of patients, together with phenotypically defined laboratory features, were classified as major criteria. Although autoimmunity and bronchiectasis have been reported in 50–70% of patients, these manifestations were regarded as supportive criteria, as they tend to occur at relatively more advanced stages of the disease, particularly in childhood (1, 3, 4, 8, 11). In patients who met at least one major clinical and one major laboratory criterion according to the diagnostic approach, genetic analysis was performed using next-generation sequencing (**Figure 1**). In patients found to carry variants of uncertain significance (VUS) according to American College of Medical Genetics and Genomics/Association for Molecular Pathology (ACMG/AMP) criteria (18), phosphorylation analysis of protein S6 was performed to confirm increased activation of the PI3K-AKT-mTOR pathway.

The study was conducted in accordance with the Declaration of Helsinki and was approved by the Local Ethics Committee of the Marmara University Faculty of Medicine (Approval Number: 15.11.2024.1301).

### Data collection

Medical history data were obtained from both electronic and paper-based medical records, as well as directly from the patients or their legal guardians. Laboratory data relevant to immunological assessment, such as complete blood count, serum immunoglobulin levels (IgG, IgA, IgM in mg/dL; total IgE in IU/mL), and detailed lymphocyte subset analysis, were collected from routine clinical evaluations that were performed at the time of the patients’ initial presentation based on medical necessity and conducted in the local laboratory. Age-appropriate reference ranges were used in the interpretation of the immunologic profile (19).

### Characterization of the identified variants

**Figure 2:**
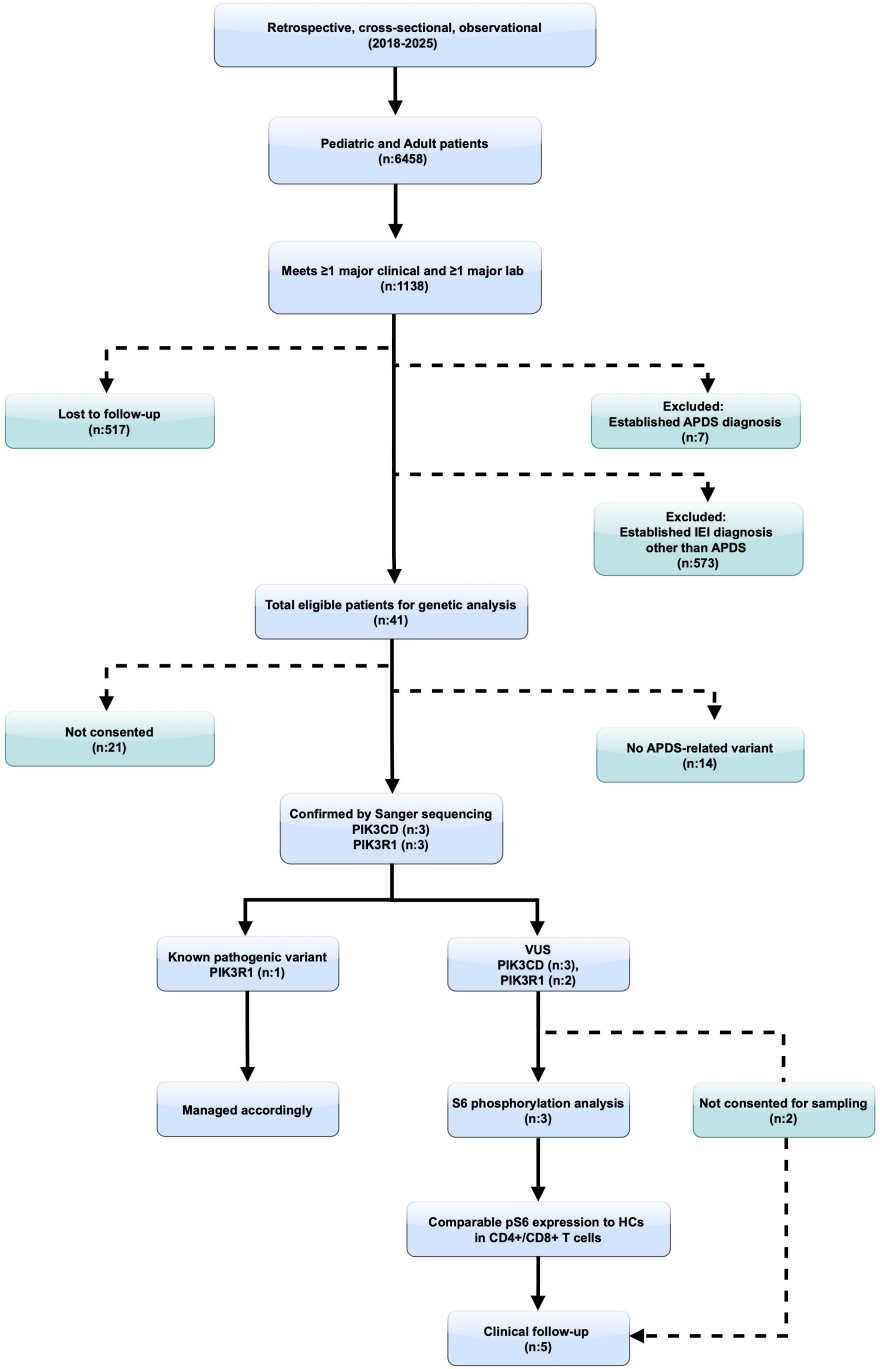
Detailed schematic of the diagnostic screening process for patients with Activated PI3Kδ Syndrome (APDS). HC, healthy controls; IEI, inborn errors of immunity; VUS, variant of uncertain significance.

Genetic evaluation was performed in a total of 20 cases (**Figure 2**). Except for one case in whom a previously reported pathogenic variant in *PIK3R1* was identified by clinical exome sequencing, whole-exome sequencing was performed in the remaining 19 cases. Variants in *PIK3CD* and *PIK3R1* were evaluated according to an autosomal dominant inheritance model, and segregation analyses were performed in variant-positive cases. To determine the disease risk associated with missense variants, in silico prediction tools such as AlphaMissense, SIFT (Sorting Intolerant from Tolerant), and MutationTaster were employed (20–22). The potential impact of splice site variants was analyzed using the SpliceAI algorithm (23). Additionally, the Combined Annotation Dependent Depletion (CADD) score was utilized to comprehensively assess the pathogenic potential of all variants (24).

### Phosphorylation analysis of protein S6

In three patients carrying a VUS in *PIK3R1* or *PIK3CD*, phosphorylation of protein S6 was analyzed according to the protocol described in a previous study (25).

Briefly, PBMCs (2 × 10^6^) were plated in 96-well plates (2 × 10^5^ cells per well, 200 µL RPMI) and incubated overnight at 37 °C. Plates were pre-coated with Mouse Anti-Human CD3 (10 µg/µL; Sigma-Aldrich, 86022706) in PBS (100 µL, 2 h). After cell seeding, Mouse Anti-Human CD28 (Clone CD28.2; BD Biosciences, 555725) was added (1 µL/well). After 24 h, PBMCs from patients and healthy donors were stained with CD4-APC (BD Pharmingen, 555349), CD8-PerCP-Cy5.5 (Clone SK1; BD Biosciences, 341050), and phospho-S6 (Ser235/236) (Cell Signaling Technology, BK2211LCST), followed by FITC-conjugated Goat Anti-Rabbit Ig (Southern Biotech, 4010-02). Flow cytometry was performed on a FACSCanto and analyzed with FlowJo software.

### Statistical analysis

Data analysis was performed using the jamovi software (version 2.3.26; the jamovi project, Australia) (26). Descriptive statistics were calculated according to the distributional characteristics of the variables. Normality was assessed using the Shapiro–Wilk test. For normally distributed variables, the mean ± standard deviation (SD) was reported; for non-normally distributed variables, the median and interquartile range [IQR, 25th–75th percentiles] were used. Categorical variables were presented as counts (n) and percentages (%).

## Results

**Figure 3:**
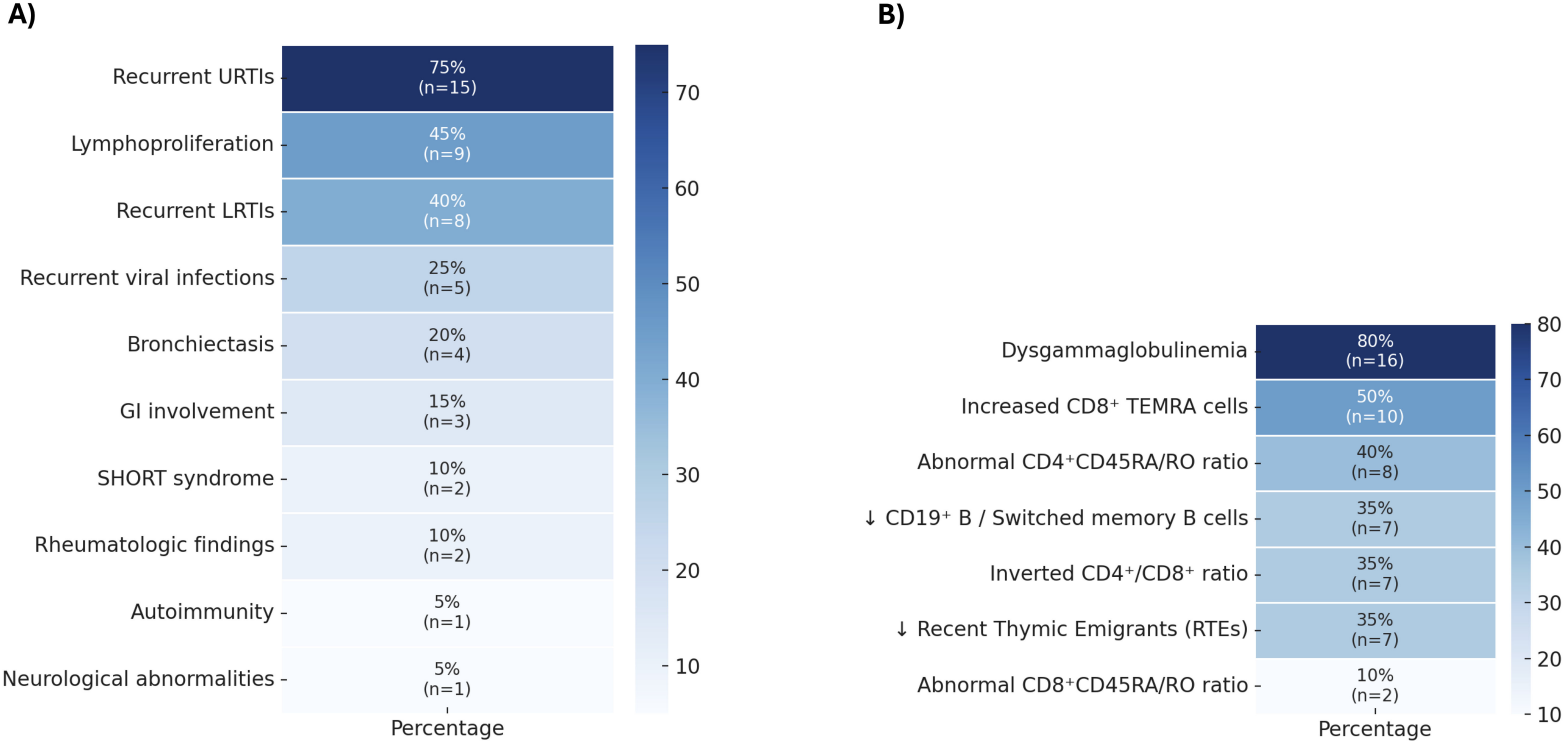
**(A)** Clinical and **(B)** immunologic profiles of patients referred for genetic analysis based on the Activated PI3Kδ Syndrome (APDS) screening strategy. I, gastrointestinal; LRTI, lower respiratory tract infection; URTI, upper respiratory tract infection; TEMRA, Terminal effector memory CCR7^-^CD45RA^+^.

From an initial cohort of 6,458 pediatric and adult patients evaluated for immune dysregulation and primary antibody deficiencies, 20 who provided informed consent and fulfilled at least one major clinical and one major laboratory criterion were selected for genetic analysis (**Figure 2**). Of these, 11 (55%) were female and 9 (45%) male, with a median age of 15 years (IQR: 7.5–24 years). A history of consanguinity was reported in 8 (40%), and 1 (5%) had a family history of an IEI. In addition to fulfilling major inclusion criteria, supportive clinical features were documented in 14 (70%), and supportive laboratory findings were observed in 9 (45%). Within this cohort, the predominant manifestations were recurrent infections, most commonly affecting the upper and lower respiratory tract, as well as viral infections. Lymphoproliferation was also frequent, whereas organ-specific complications such as bronchiectasis, gastrointestinal involvement, rheumatologic manifestations, autoimmunity, and neurological abnormalities occurred less often. Notably, two individuals were diagnosed with SHORT syndrome (**Figure 3A**).

Laboratory immune profiling demonstrated abnormalities across immunoglobulin, T-cell, and B-cell compartments (**Figure 3B**). Dysgammaglobulinemia was the most common finding, present in the majority of the cohort. T-cell abnormalities were also frequent, including skewed CD4^+^ and CD8^+^ subsets, an inverted CD4^+^/CD8^+^ ratio, and reduced levels of Recent Thymic Emigrants. B-cell analysis revealed impaired maturation, reflected by decreased switched memory B cells.

Genetic analysis identified rare variants in APDS-associated genes in six individuals—three in *PIK3CD* and three in *PIK3R1*. Notably, P2 carried a previously reported splice-site variant in *PIK3R1* (c.1425 + 1G>C) (10), which was deemed causally relevant to the disease phenotype. Family segregation analysis confirmed the absence of this variant in first-degree relatives, consistent with a *de novo* occurrence. The patient’s management was directly informed by this established genetic etiology: IgRT and trimethoprim–sulfamethoxazole prophylaxis were initiated for infection prevention, and sirolimus therapy was subsequently introduced following exclusion of malignancy by excisional cervical lymph node biopsy performed for persistent lymphadenopathy.

**Figure 4:**
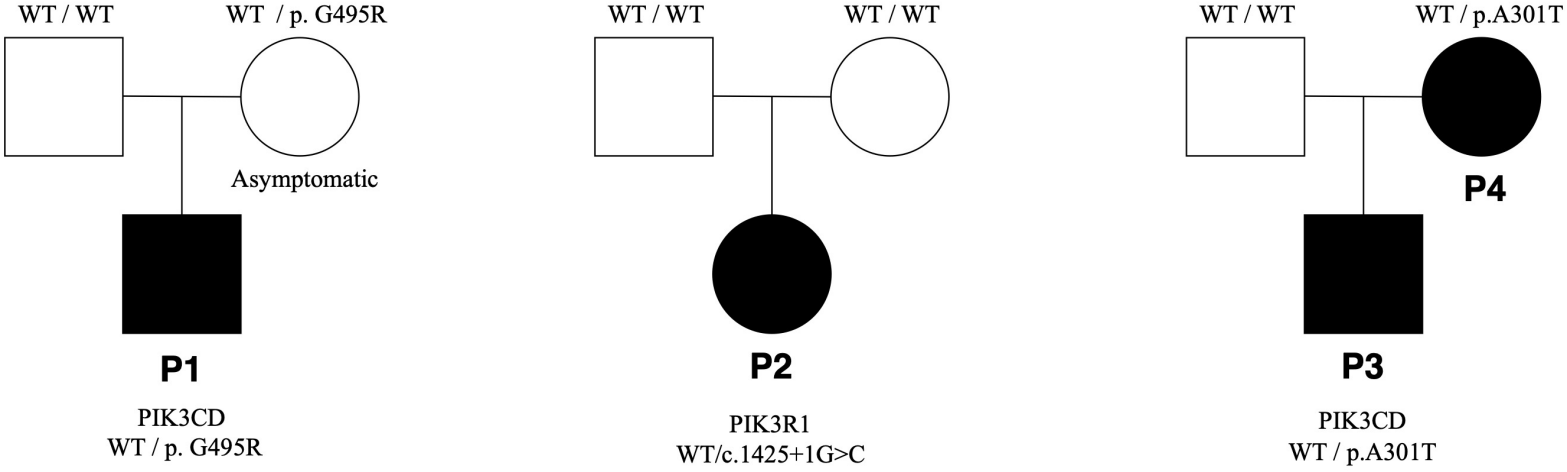
Pedigrees of the patients. P, patient; WT, wild type.

The remaining five individuals carried novel missense variants in *PIK3CD* (p.G495R; p.A301T [n=2]) and *PIK3R1* (p.G599R; p.M326L). All were classified as VUS according to ACMG criteria (**Table 1**). In silico prediction algorithms yielded variable results, with some suggesting potentially deleterious effects. Clinically, these individuals manifested heterogeneous but overlapping features of immune dysregulation—most prominently recurrent respiratory tract infections, lymphoproliferation, and dysgammaglobulinemia—sometimes accompanied by additional systemic findings. While these observations point to a possible functional contribution of the identified variants, definitive evidence for pathogenicity is lacking at present. Family segregation analysis was performed for all cases except P5 and P6. In these two affected adults, parental testing was not possible; therefore, family segregation analysis could not be completed. Notably, the mother of P1 carried the same *PIK3CD* variant as her child yet remained asymptomatic, with normal immunological parameters on screening (**Figure 4**).

**Table 1:** Clinical, laboratory, and genetic features of six patients with variants detected in the APDS gene as a result of screening.

Patients	P1	P2	P3	P4	P5	P6
Family	F1	F2	F3	F3	F4	F5
Current age (years)	19	11	5	28	28	54
Gene	PIK3CD	PIK3R1	PIK3CD	PIK3CD	PIK3R1	PIK3R1
Variant	c.1483G>A p. G495R	c.1425 + 1G>C	c.901G>A p.A301T	c.901G>A p.A301T	c.1795G>C p.G599R	c.976A>T p.M326L
Novel Variant	Novel	Reported	Novel	Novel	Novel	Novel
Inheritance	AD	AD	AD	AD	AD	AD
AF (%) (gnomAD, global)	0.000502%	0 (not observed)	0 (not observed)	0 (not observed)	0 (not observed)	0.000398%
Variant type	Missense	Splice site	Missense	Missense	Missense	Missense
ACMG	VUS	Pathogenic	VUS	VUS	VUS	VUS
AlphaMissense	Pathogenic Supporting (0.822)	–	Benign Strong (0.063)	Benign Strong (0.063)	Uncertain (0.544)	Benign Moderate (0.11)
SIFT	Uncertain (0.032)	–	Benign (Moderate) (0.427)	Benign (Moderate) (0.427)	Uncertain (0.002)	Benign (Moderate) (0.408)
MutationTaster	Deleterious (1)	Deleterious (1)	Benign (0)	Benign (0)	Deleterious (1)	Deleterious (1)
SpliceAI	–	Strong (Δ score: 1)	–	–	–	–
CADD score	23	34	2.68	2.68	27.6	22.5
Clinical and Laboratory Features	Recurrent URTI, lymphoproliferation, dysgammaglobulinemia, impaired antibody response to antigens.	Recurrent URTI, lymphoproliferation, dysgammaglobulinemia, impaired antibody response to antigens, inverted CD4+/CD8+ ratio.	Recurrent URTI and LRTI, truncus arteriosus, epilepsy, dysgammaglobulinemia, impaired antibody response to antigens, inverted CD4+/CD8+ ratio.	Recurrent URTI, dysgammaglobulinemia.	Recurrent URTI, meningitis, dysgammaglobulinemia.	Recurrent URTI and LRTI, bronchiectasis, Addison’s disease, dysgammaglobulinemia, ↓CD19^+^ B cells, inverted CD4/CD8 ratio, ↑CD8^+^CD45RA^+^CD27^-^ TEMRA cells, abnormal CD4^+^CD45RA/RO ratio.

ACMG, American College of Medical Genetics and Genomics; AD, Autosomal Dominant; AF, Allele frequency; APDS, Activated Phosphoinositide 3-Kinase Delta Syndrome; CADD, Combined Annotation Dependent Depletion; F; Family; LRTI, Lower Respiratory Tract Infection; P; Patients; SIFT, Sorting Intolerant from Tolerant; URTI, Upper Respiratory Tract Infection; VUS, Variant of Uncertain Significance.

Functional assessment of S6 phosphorylation was conducted in P1, P3, and P4, as well as in P1’s mother; however, it could not be performed in P5 and P6 due to lack of consent.

While P1 showed increased pS6 levels both in CD4^+^ and CD8^+^ T cells upon *in vitro* activation, P1’s mother harboring the same *PIK3CD* variant did not exhibit signs of PI3K hyperactivation, suggesting that the former maybe due to external factors such as recent or ongoing viral infection or similar (**Supplementary Figures 1B, C**). Evaluation of pS6 levels in CD4^+^ and CD8^+^ T cells upon activation from P3 and P4 did not reveal any increase in the activation of the PI3K pathway (**Supplementary Figures 1B, C**).

To further explore the contribution of genetic etiologies, whole-exome sequencing was performed but did not reveal additional variants that could account for the patients’ clinical or immunological phenotypes.

## Discussion

In this study, we applied a diagnostic approach, developed based on existing literature and our clinical experience, to a large cohort (n = 6,458) evaluated for primary antibody deficiencies and immune dysregulation. Six APDS-associated variants were identified, and one patient received a definitive diagnosis. Unlike previous studies, this study conducted systematic screening in a broad and heterogeneous patient population, thereby providing both genetic findings and practical insights into the diagnostic process (3, 7). Additionally, the challenges encountered during diagnosis were described in detail, and these observations were translated into actionable recommendations aimed at improving clinicians’ ability to achieve early diagnosis.

In our study, the prevalence of APDS was 1 in 6,458. Since it was first described in 2013, over 350 cases of APDS have been documented, although the true prevalence remains unknown (15, 27–29). Current estimates suggest an occurrence of approximately 1–2 cases per million (2). The higher prevalence observed in our study, compared to general population estimates, may be attributed to both the implementation of systematic screening and the selection of a cohort with primary antibody deficiencies and immune dysregulation. These findings indicate that targeted genetic screening can substantially improve diagnostic yield, particularly in high-risk patient populations.

In the patient harboring the pathogenic *PIK3R1* variant, recurrent respiratory tract infections began at approximately 1 month of age, with splenomegaly and hepatomegaly developing at 10 years of age. APDS typically presents in early childhood, with a median onset age of about 1 year, and is characterized by recurrent, severe respiratory tract infections (1, 5, 30). Benign lymphoproliferation is common in the early stages, while advanced stages may involve complications such as enteropathy, autoimmune diseases, bronchiectasis, and malignant transformation. Persistent or chronic infections with herpesviruses (e.g., EBV, CMV, HSV) are frequent and can exacerbate the disease (2, 3, 8, 12, 30). Taking this clinical picture into account, in our study we focused on the most frequent manifestations occurring in the early stages of the disease when defining the major criteria. Although autoimmune manifestations are common, because they usually appear at more advanced stages of the disease during childhood, the presence of autoimmunity alone in the absence of a history of recurrent infections was regarded as a supportive rather than a major criterion (1, 3, 4, 8, 11). On the other hand, given that the median age in our selected cohort was 15 years, considering autoimmunity as a supportive criterion may have partially limited the recognition of adult patients. The expression of PI3Kδ in non-immune tissues suggests that APDS can also affect other organs and systems (2, 6, 31). Neurodevelopmental abnormalities, short stature, and growth retardation, though not present in every patient, are more common in APDS2 (*PIK3R1* LOF) cases. In our patient, no neurodevelopmental abnormalities or short stature were observed. In our selected cohort, evaluated for primary antibody deficiencies and immune dysregulation to screen for APDS, the most frequent findings were recurrent respiratory tract infections (75%) and lymphoproliferation (45%). APDS-associated variants were absent in 70% of patients. The wide and variable presentation of APDS, along with its overlap with other immune disorders, makes diagnosis challenging. Careful recognition of distinctive clinical features and confirmation by genetic testing are essential for accurate diagnosis and appropriate treatment (2, 8).

The PI3Kδ enzyme complex plays a pivotal role in immune regulation, particularly through its expression in leukocytes (2, 6, 32). Gain-of-function variants in the *PIK3CD* gene and loss-of-function pathogenic variants in the *PIK3R1* gene lead to hyperactivation of the PI3K–Akt–mTOR–S6 signaling pathway in B and T lymphocytes (9, 33, 34). In B cells, this hyperactivation results in lymphopenia, reduced numbers of naïve and memory B cells, and impaired class-switch recombination, ultimately causing inadequate humoral responses. Consequently, low serum IgG and IgA levels, along with elevated IgM levels, may be observed. Increased frequencies of transitional B cells and CD21^low^CD38^low^ B cells have also been reported (2–4, 8, 9, 11). In T cells, PI3K hyperactivation is associated with a decrease in CD4^+^ T lymphocytes and an increase in CD8^+^ T lymphocytes, leading to an inverted CD4^+^/CD8^+^ ratio. Additionally, increased effector T-cell differentiation and the accumulation of senescent T cells (CD57^+^CD3^+^) have been reported (2, 4, 8, 9, 11). Based on these immunological features, our screening strategy defined major criteria as dysgammaglobulinemia, decreased CD19^+^ B cells/decreased CD19^+^CD27^+^IgM^-^IgD^-^ cells, an inverted CD4^+^/CD8^+^ T cell ratio, and increased CD8^+^CD45RA^+^CD27^+^ cells. The APDS type 2 patient identified through this screening also exhibited dysgammaglobulinemia, impaired antibody responses to antigens, and an inverted CD4^+^/CD8^+^ ratio.

A limitation of our study is the lack of systematic assessment of APDS-associated B-cell subpopulations. Because transitional B cells and CD21^low^ B cells are not routinely included in diagnostic flow cytometry panels for all patients, we were unable to analyze these subsets as supportive laboratory criteria. Additionally, further studies are needed to support the consistent inclusion of autoimmunity as a major rather than a supportive clinical feature in APDS screening frameworks. Standardized inclusion of these markers and harmonized definitions of major criteria in future studies may enhance the clinical utility of screening approaches.

In our study, next-generation sequencing identified VUS in the *PIK3CD* gene in patients P1, P3, and P4, and in the *PIK3R1* gene in patients P5 and P6. Considering the possibility of asymptomatic or mildly symptomatic individuals within the family, segregation analysis was performed for all patients (2, 5). In P1’s family, the same variant was detected in his asymptomatic mother. Although APDS patients typically exhibit high penetrance and this finding argues against the diagnosis, the possibility of APDS cannot be entirely excluded when considering the clinical features and variant pathogenicity (2, 3, 11, 35). Despite the low pathogenicity of the variants in P3 and P4, their clinical findings were suggestive of APDS. In these three patients, intracellular phosphorylation analysis of basal or activated protein S6 in CD4^+^ and CD8^+^ T cells were performed to assess activation of the PI3K–AKT–mTOR pathway; however, no pathological increase in S6 phosphorylation was detected. In P3 and P4, the combination of low variant pathogenicity and the absence of increased S6 phosphorylation did not support a diagnosis of APDS. In contrast, in P1, despite no evidence of pathological increase in S6 phosphorylation, in silico predictors suggested a deleterious effect of the variant, making it a strong candidate to explain the clinical presentation. AKT and S6 phosphorylation assays are available only at select centers and may be affected by sample transport conditions (2, 9, 11). Therefore, the absence of increased phosphorylation does not definitively exclude the diagnosis. In patients with a phenotype compatible with APDS but without confirmed variant pathogenicity, continued clinical follow-up is important, as information in the literature and variant databases continues to evolve (2, 36). Accordingly, follow-up is ongoing for all our patients.

In conclusion, APDS is a rare inborn error of immunity marked by recurrent infections and lymphoproliferation. Early recognition of its clinical features is essential to prevent irreversible organ damage and enable timely intervention. Due to its broad and heterogeneous phenotype, which often overlaps with other primary immunodeficiencies, genetic testing is critical for establishing a definitive diagnosis. In cases where VUS are identified, functional analyses should be performed, and patients must be closely monitored over time. With the increasing availability of targeted therapies, early diagnosis and genetic confirmation are vital for improving clinical outcomes and quality of life in affected individuals.

## Data Availability

The original contributions presented in the study are included in the article/**Supplementary Material**. Further inquiries can be directed to the corresponding author.

## Supplementary material

Supplementary Figure 1 Evaluation of pS6 levels in T cells upon activation. (A) Gating strategy from a healthy donor to define CD4^+^ and CD8^+^ T-cell subsets and assess pS6 levels. (B) Representative histograms of pS6 in CD4^+^ and CD8^+^ T cells before (gray) and after (white) in vitro activation in a healthy donor (HD) and patients P1, P1’s mother, P3, and P4; numeric labels indicate mean fluorescence intensity (MFI). (C) Summary data of pS6 levels after in vitro activation for the index patients and an HD cohort (n = 11). HD, healthy donor; MFI, mean fluorescence intensity; pS6, phosphorylated S6.
