# Supplementary figures and images for: Activated PI3Kδ syndrome in inborn errors of immunity: diagnostic strategies and clinical challenges
Source: Front Immunol. 2026 Jan 8;16:1735023. doi: 10.3389/fimmu.2025.1735023 (PMC12823944; doi:10.3389/fimmu.2025.1735023)

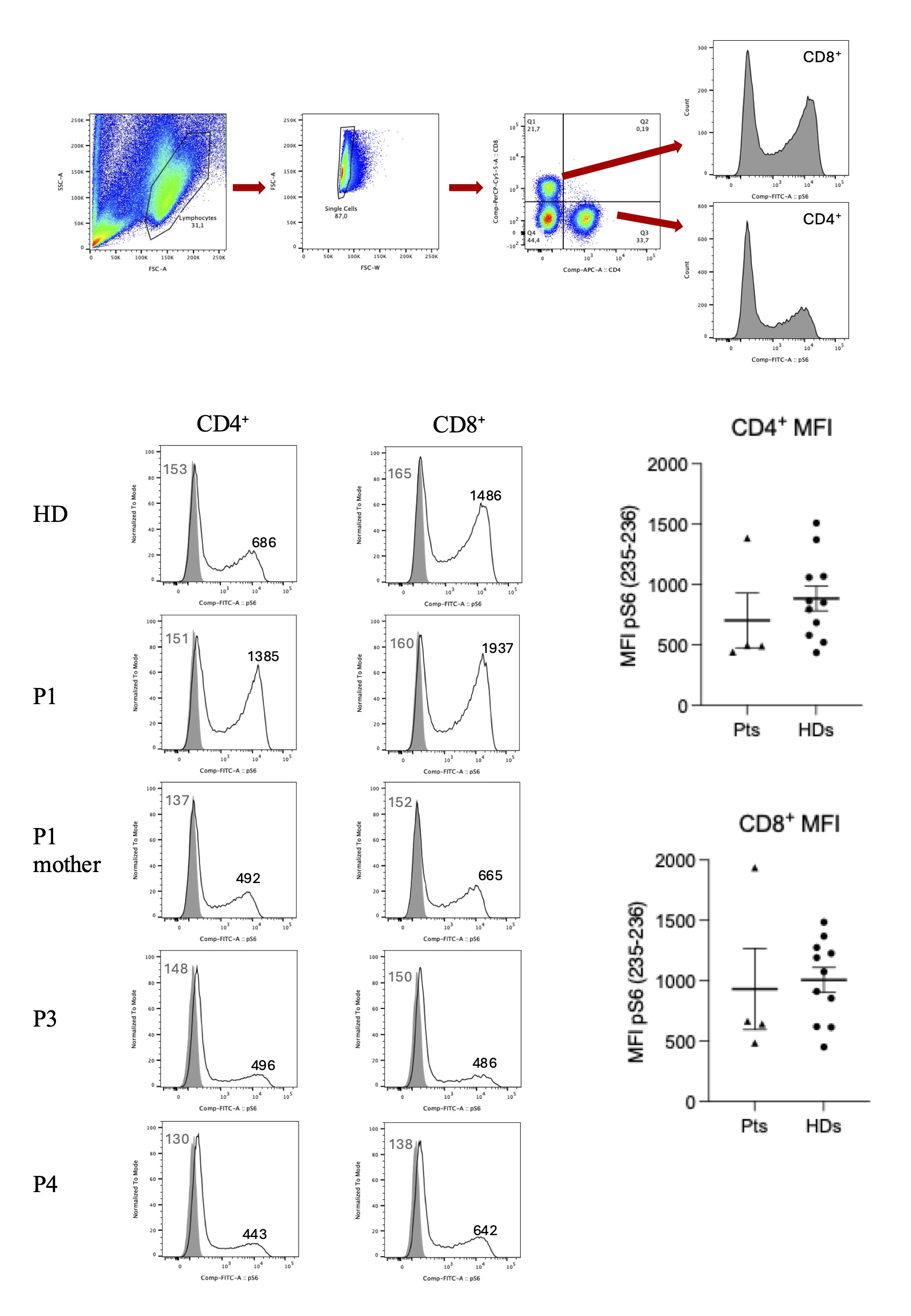

Supplement: Supplementary Figure 1 — Evaluation of pS6 levels in T cells upon activation. (A) Gating strategy from a healthy donor to define CD4+ and CD8+ T-cell subsets and assess pS6 levels. (B) Representative histograms of pS6 in CD4+ and CD8+ T cells before (gray) and after (white) in vitro activation in a healthy donor (HD) and patients P1, P1’s mother, P3, and P4; numeric labels indicate mean fluorescence intensity (MFI). (C) Summary data of pS6 levels after in vitro activation for the index patients and an HD cohort (n = 11). HD, healthy donor; MFI, mean fluorescence intensity; pS6, phosphorylated S6. [file Image1.jpeg]
